# Role of EEG in Measuring Cognitive Reserve: A Rapid Review

**DOI:** 10.3389/fnagi.2020.00249

**Published:** 2020-08-26

**Authors:** Kristı̄ne Šneidere, Sara Mondini, Ainārs Stepens

**Affiliations:** ^1^Military Medicine Research and Study Centre, Rı̄ga Stradiņš University, Riga, Latvia; ^2^Department of Health Psychology and Paedagogy, Rı̄ga Stradiņš University, Riga, Latvia; ^3^Department of Philosophy, Sociology, Pedagogy and Applied Psychology, University of Padua, Padua, Italy

**Keywords:** cognitive reserve, EEG, event-related potentials, resting-state EEG, relaxed-state EEG

## Abstract

This review aimed to systematically summarize the possible neural correlates of cognitive reserve thus giving an insight into prospective biomarkers for the concept. A total of 44 studies were analyzed following PRISMA guidelines and four studies were included in the further analysis. The results indicated a relationship between P3b waveform and cognitive reserve, while more ambiguous outcomes were found when conducting resting-state EEG. This review indicates the first steps into assessing CR using physiological measures; however, more research is needed for deeper understanding of its underlying mechanisms.

## Introduction

With the rapid increase of the availability of brain integrity measuring techniques, such as magnetic resonance imaging (MRI) or scalp electroencephalogram (EEG), the interdisciplinarity between cognitive psychology and neurology (i.e., cognitive neuroscience) has become stronger and the data—more reliable. So far there have been several attempts to try to identify an objective measure of cognitive reserve (CR)—one's ability to adapt better to brain pathology or age-related cognitive changes (Stern et al., 2018)—using functional MRI (fMRI), magnetoencephalography (MEG) and EEG; however, a specific objective measure has yet to be confirmed. The recent studies have identified event-related potentials (ERPs) and resting-state EEG as the possible objective measures for cognitive processes, as well as CR.

### Cognitive Reserve

CR indicates the efficiency, capacity, and flexibility of cognitive processes in the presence of a challenge, which helps to explain the individual's ability to cope better with brain pathology (e.g., brain aging, delay of dementia symptoms, stroke) via more adaptable functional brain processes (Stern et al., 2018). Cognitive reserve is thought to be measured with different proxies—education, verbal IQ, occupational activity, social, and leisure activities, etc. (Lojo-Seoane et al., 2014; Stern, 2017). Nowadays the most informative measurement for cognitive reserve are composite measures—an index that includes different aspects of CR, e.g., Cognitive Reserve Index questionnaire, that comprises of education, occupational attainment, and leisure time activity (Nucci et al., 2012).

While the actual biomarkers of CR are still questioned, a possible mechanism for CR has been hypothesized. Neural reserve theory postulates that there exists an interindividual variability in brain networks that function as a basis of any task. If an individual meets a specifically challenging task, they engage neural reserve in completing the task more efficiently, i.e., neural activity would request lesser energy consumption. The idea of neural compensation is that individual who suffers from brain pathology (e.g., Alzheimer's disease or traumatic brain injury) can engage other, unrelated, but relatively intact, brain structures or networks that are normally not used for the specific performance. Depending on the demand, the alternate network or brain structure can be used periodically (Steffener and Stern, 2012).

### Brain Reserve

Brain reserve (BR) concept refers to the neurobiological capital of an individual, e.g., number of neurons and synapses that enables them to better cope with aging or pathology (Stern et al., 2018). Stern et al. (2019) characterizes BR as a physical trait that allows individuals to tolerate more significant loss of neural substrate due to larger brain, thus delaying or lessening the predicted cognitive impairment due to, e.g., neurodegenerative disease or stroke.

Literature often contains a misunderstanding of both concepts—CR and BR. If CR overall could be defined as a learnt competency of using ones' brain, BR indicates the physical characteristics of the brain, e.g., volume or synaptic count (Tucker and Stern, 2011). Considering both concepts it is important to note that CR is essentially limitless, while BR has a fixed threshold. Stern characterizes the difference between the two concepts using the term “software” regarding CR and “hardware” when talking about BR, which might give the impression of CR being strictly about the functionality and BR being only about the physical measures; however, it should be taken into consideration that CR undoubtedly has a neuronal counterpart (Stern, 2017). This idea has been supported by functional MRI studies, though there is still a lack of conclusive results regarding the specific neural networks or regions involved (Stern et al., 2018). Overall, this indicates the tendency to focus on functionality when considering CR, and volume when considering BR; therefore, EEG studies could be beneficial in investigating neural aspects of EEG.

Using EEG is a way of exploring networks and functionality. Originally developed for examining patients with epilepsy, currently, it can be used for a wide variety of reasons, including investigating functional connectivity and functional brain networks, thus allowing the assessment of cognitive processes. Scalp EEG is a non-invasive, rather inexpensive, and easily movable method for assessing neurophysiological functions via measuring the electrical activity from electrodes planted on the scalp (Light et al., 2010).

The aim of the study is to systematically summarize the possible neural correlates of cognitive reserve measured with EEG in adults.

## Materials and Methods

### Literature Search

This rapid review was conducted in accordance with PRISMA guidelines. A systematic search in the electronic databases Scopus and Web of Science was performed, using the keywords “cognitive reserve AND event-related potentials” and “cognitive reserve” AND EEG.

### Eligibility Criteria

Only the studies in English and published from 2009 to 2019 were included in the search. We included the studies that reported either standardized questionnaire for measuring CR or a composite score consisting of a combination of different proxies of CR and excluded the studies that used only one proxy of CR (e.g., either education or verbal IQ or working activity), as well as only the studies reporting human adult samples were included. Reviews, systematic reviews, and meta-analysis were excluded.

### Study Selection

The papers were identified according to the eligibility criteria. Firstly, the abstracts of the articles were obtained and inspected, secondly, the method descriptions were thoroughly investigated to determine the score used for CR.

## Results

**Figure 1:**
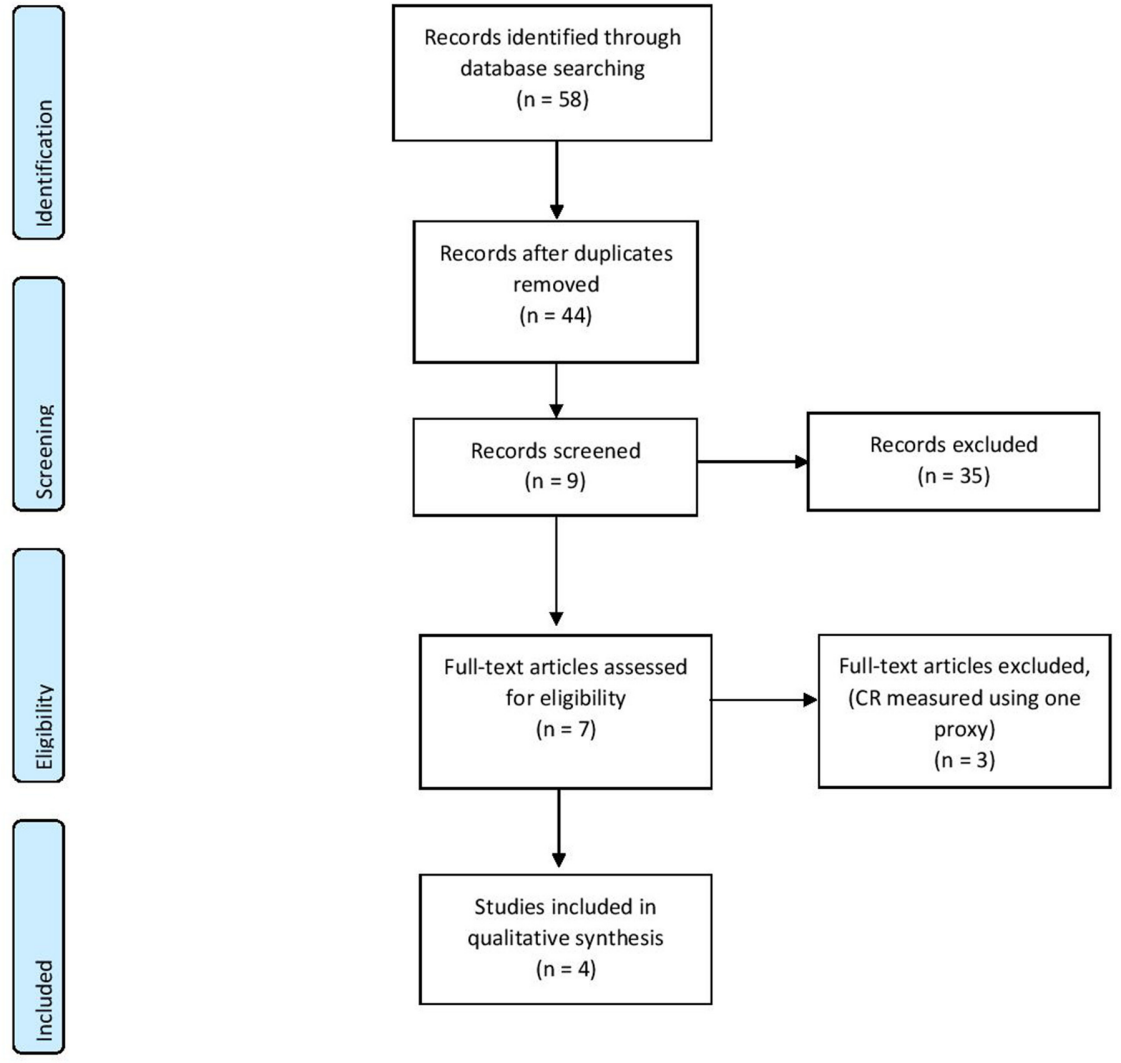
PRISMA diagram of the reviewing process (Moher et al., 2009).

Based on the keywords, 58 records were identified through database searching, and after duplicate removal, 44 studies were selected for the first screening. After applying the eligibility criteria to the abstracts, only seven full-text articles were deemed to fit and full texts were assessed for eligibility. After full-text screening, only four studies were included in the qualitative synthesis (see Figure 1). These studies investigated two types of EEG measures—event-related potentials and resting-state EEG.

So far, two papers have been published examining the relationship between composite cognitive reserve score (i.e., a proxy for CR consisting of several measures) and P300 waveform (see Table 1).

**Table 1:** Results after qualitative synthesis.

**References**	**Participants**	**CR measure**	**EEG measure**	**Number of channels**	**Results**
Speer and Soldan (2015)	Healthy young and older adults (25:21)	A composite score of results of language tasks and education	P300b	32 scalp ActiveTwo electrodes, Biosemi	Higher CR associated with lower neural inefficiency
Gu et al. (2018)	Healthy older adults and older adults with MCI	CRIq	P300	64 scalp electrodes	Different networks used in healthy vs. aMCI groups
Amodio et al. (2017)	82 patients with liver cirrhosis	CRIq	EEG traces in P3-P4 derivation	21 Ag/AgCl electrodes	No significant relationships between CRIq and physiological parameters
Fleck et al. (2017)	90 healthy adults (*M* = 58.51)	A composite score of verbal IQ and education	Frequency bands: delta, theta, low alpha, high alpha, beta, gamma	129-channel HydroCel Geodesic Sensor Net	Significant decrease in resting-state coherence with aging in low-CR

One of the first studies considering ERPs was conducted by Speer and Soldan (2015). Forty-four participants (25 healthy young adults and 21 non-demented healthy older adults) were involved in the study. CR was measured creating a composite score of National Adult Reading Task, vocabulary subtest of Wechsler Adult Intelligence Scale-Revised, and years of education. As a measure of cognitive performance, a delayed item recognition task was used. This task was focused on measuring verbal working memory. To determine neural efficiency and neural capacity, brain electrical activity was recorded from 32 scalp sites and a specific type of P300 (P3b) was measured.

The association between CR and neural efficiency was investigated using a CR composite score and an index of neural inefficiency (ratio of the amount of demand-related neural activity, also—P3b amplitude slope with increasing task load). Results indicated that higher composite CR was associated with a better neural efficiency in both—accuracy and reaction time.

Neural capacity, which was determined based on P3b changes in amplitude and latency with increasing task difficulty, was increased in younger adults, but not in older. Neural capacity indicates higher network expression at higher levels of task demand, and such results demonstrate that younger participants could boost activation when the tasks became more challenging.

A more recent study was conducted by Gu et al. (2018). They aimed to investigate and compare the effect of CR on brain activation in two groups—healthy controls and patients with amnestic mild cognitive impairment (aMCI). Overall 85 participants were included in the study (46 controls and 39 aMCI patients) aged from 55 to 80 years. The participants underwent neuropsychological assessments considering general cognitive functioning, episodic memory, executive function, information processing speed, and visual-spatial domains. Cognitive Reserve Index questionnaire (CRIq) (Stern et al., 2019) was used as a measure of cognitive reserve and brain activation measures were recorded from 64-electrode scalp sites. The N-back task was used as task performance. The N-back task involved two types of tasks−0- and 1-back. In the 0-back task, the participants had to recognize whether the white block appeared on the upper left side of the screen, while in 1-back task participants had to determine whether the displayed stimulus had already been displayed at the same locations of the previously presented one. Accuracy and response time were recorded as well. P300 waveform was used for neural inefficiency calculations and was assessed at central-parietal and parietal electrodes.

Results indicated that neural inefficiency partially mediated the association between CR and task performance in healthy seniors and that higher CR reduced neural inefficiency; however, no such relationship was discovered in the aMCI patient group. Interestingly, it was found that in attention processes healthy controls used different networks than the aMCI group.

Amodio et al. (2017) opted to assess the role of CR on the cognitive expression of covert hepatic encephalopathy recording participants in a condition of relaxed wakefulness. Eighty-two outpatients (*M*_age_ = 62, 54–68, 73% male) with liver cirrhosis in a stable condition and no overt hepatic encephalopathy participated in the study. Neuropsychological assessment was conducted using the PSE syndrome test, which includes five tasks and measures of attention, executive functions, and visual- and psychomotor abilities. As in Gu et al., CR was measured using Cognitive Reserve Index questionnaire (Nucci et al., 2012; Gu et al., 2018). As a neurophysiological measure, 10 min EEG was recorded in a condition of relaxed wakefulness, using a standard cap with 21 Ag/AgCl electrodes.

Data indicated two main results. Firstly, the patients with higher cognitive reserve also related to better results in neuropsychological tasks, especially, executive functioning measures. Secondly, EEG measures did not show any relationship with cognitive reserve.

Another study by Fleck et al. (2017) was published in the same year. Fleck and colleagues aimed to investigate the differences in resting-state connectivity in relation to CR using EEG. Ninety healthy volunteers (*M*_age_ = 58.51, *SD* = 4.37, 66% female) with no self-reported history of traumatic brain injury, stroke, neurological disorders, two or more concussions, history of drug and alcohol abuse, current use of medicine for treatment for anxiety or depression and MMSE score under 26. All participants were divided into two groups depending on the cognitive reserve level (*n*_low_ = 43 and *n*_high_ = 47). CR score was estimated from verbal IQ score and years of education. EEG measures were conducted with 129-channel Hydrogel Geodesic Sensor Net with Cz reference. The participants were asked to sit 3 min with eyes open and 3 min with eyes closed. In addition, several neuropsychological measures were conducted, including general cognitive assessment using MMSE-2, visuospatial abilities, verbal intelligence, memory, and executive functioning.

Results indicated a negative correlation between age and brain coherence in low-CR participants, while in high-CR participants it was positive. Results also showed global coherence differences between age groups for left and right hemisphere connectivity and between cognitive reserve groups for eyes-closed and eyes-open recording conditions.

## Discussion

In this review, we aimed to investigate and summarize the possible neural correlates of cognitive reserve (CR) as measured with EEG. The results indicated that there has been little research done using a composite measure of CR and EEG.

While the focus of this paper is not on brain reserve (BR), it is understandable that both concepts are not separate, but rather two sides of the same coin, therefore, investigation of possible neuronal correlates is important. This is supported by the increasing use of EEG in mild cognitive impairment and Alzheimer's disease investigations, showing that even subtle brain changes can be identified in the patients with MCI in comparison to normally aging individuals (Rossini et al., 2008; Babiloni et al., 2016), possibly—prior to manifestations of behavioral and cognitive symptoms, which can often be delayed by the protective nature of CR.

Two of the studies measured event-related potentials, specifically, P300 waveform. P300 is often considered to be a biomarker for cognitive functioning (Sur and Sinha, 2009). While the results showed differences in low- and high-CR groups, it could still be related to better cognitive functioning rather than indicate CR. Especially considering that in both studies, the given tasks are traditionally used in working memory studies [see Kirchner for n-back task and Sternberg for Sternberg working memory task Kirchner, 1958; Sternberg, 1966], changes in P300 could be a biomarker for working memory. This corresponds to even older studies that have indicated a relationship between increase in P300 waveform and working memory load (Dolu et al., 2005). At the same time, while there are few studies confirming the correlation between cognitive functioning and CR, a recent finding by Lojo-Seoane et al. (2020) indicate that working memory might be the main underlying cognitive function that mediates the connection between CR and cognitive performance. Therefore, even though the aforementioned studies used P300 as a prospective marker for CR, it is still possible that it is an indicator of working memory functioning as a mediator.

Whereas ERP studies had seemingly similar outcomes, the results from the resting-state studies had less unanimous conclusions, with one study indicating no significant relationship between CR and brain waves measured with scalp EEG, and another showing dissimilarities in brain hemisphere coherence in people with different CR levels. While the results from the studies reviewed within this article do not provide conclusive evidence regarding CR, studies that use resting-state EEG in cognitive neuroscience research show contrasting results with some researchers claiming that resting-state EEG could function as a screening method for cognitive decline [e.g., see (Choi et al., 2019)], thus resting-state EEG could be considered a neural condition reflecting cognitive decline rather than a biomarker itself.

Another considerable aspect is that not a single measure of CR was used. While only composite scores were used as proxies for CR, the distribution of elements considered to be building blocks of CR was not found in all proxies. For example, occupational activity has been found as a significant component of CR (Adam et al., 2013), but it is excluded from the composite score in two of the reviewed studies. Similarly, while CRIq offers a detailed examination of the life-time activities, it lacks information on verbal IQ—another component considered to be a significant identifier of CR (Leoń et al., 2014). A meta-analysis by Opdebeeck et al. (2016) showed that different proxies of CR yield different relationship with specific cognitive functions, e.g., the strength of correlations between leisure-time related CR and working memory is lower than it is when using education as a proxy. The optimal choice could be combined CR measures; still such data are often obtained by retrospective questionnaires and thus rely mostly on patients or caregivers memories.

The aforementioned argument extends not only to the understanding of CR and identifying life-style proxies, but also to the understanding of the mutual relationship between CR and BR. While all articles included in the review note that they essentially aim to confirm EEG markers as biomarkers for CR, it should be carefully considered whether ERPs and brain waves can be defined as measuring specifically CR rather than BR. While there are several studies using fMRI, the results are still vague and does not offer a conclusive map of either cognitive or brain reserve (Stern et al., 2018).

The major limitation of the review was the small sample of reviewed articles. While study results indicate promising work toward the use of EEG in investigating CR, it should be taken into consideration that only four studies met the inclusion criteria. This review has only considered articles using composite measures of CR, which has presented as both—a limitation and a strength of the study. As discussed above, three different measures of CR were used, therefore the results are not directly comparable.

This review evaluated in detail the potential of EEG in investigating CR. To our knowledge, this is the first review to examine the use of EEG in combination with CR. The findings indicate that further investigations of EEG use in CR research would be useful, as EEG is a relatively inexpensive and more accessible way of measuring CR. While it is clear that CR is a multidimensional concept and cannot be measured by a single biomarker, further studies are necessary for deeper understanding of the underlying neural mechanisms.

## Author Contributions

KŠ and AS contributed to the conception and design of the review and wrote the first draft of the manuscript. KŠ organized the database. SM contributed to specifying the theoretical concept. All authors contributed to manuscript revision, read, and approved the submitted version.

## Conflict of Interest

The authors declare that the research was conducted in the absence of any commercial or financial relationships that could be construed as a potential conflict of interest.
